# How does urban amenity affect the willingness of college youth to stay in the city?—empirical evidence from Chinese first-tier cities

**DOI:** 10.3389/fpubh.2025.1661100

**Published:** 2025-10-22

**Authors:** Zhaowang Chen, Junna Liu

**Affiliations:** ^1^College of Philosophy, Law and Political Science, Shanghai Normal University, Shanghai, China; ^2^School of Public Administration, University of Electronic Science and Technology of China, Chengdu, Sichuan, China

**Keywords:** urban amenity, youth in colleges, talents, willingness to stay in the city, scale of urbanamenity

## Abstract

**Introduction:**

For countries in the process of transition from a traditional economy to a knowledge-based and innovative economy, talent is the key to ensure the success of the transition. As the main source of talents, youth in colleges are the foundation and important driving force of social and economic development, and their willingness to stay in the city is of great significance to the development of the city. Previous studies have shown that urban amenity is the attraction of the city to talents, and this study aims at exploring the impact of urban amenity on the willingness of youth in colleges to stay in the city.

**Methods:**

First, based on the scientific scale development process, we developed the urban amenity scale based on the subjective evaluation of urban residents. Second, through questionnaire survey, the developed scale was used to verify the positive impact of urban amenity on the willingness of youth in colleges to stay in the city. And the binary logit model was employed in this study.

**Results:**

This study finds that urban amenity positively affects the willingness of colleges youth in first-tier cities to stay in the city. The three dimensions of urban amenity - urban work amenity, urban life amenity, urban cultural amusement and study amenity - all positively affect the willingness of college youth to stay in the city. Moreover, by comparing the Odd Ratio of college youth’s willingness to stay in the city in first-tier cities, it is found that urban cultural amusement and study amenity has the greatest impact on the willingness of college youth to stay in the city, second is urban life amenity, and the last is urban work amenity.

**Discussion:**

The findings of this study add nuance to the global literature by demonstrating that for Chinese college youth, urban amenities are not a replacement for economic concerns but a complement. The significance of all three dimensions of amenity shows that Chinese talents seek a “complete package”. It means a city that should offers strong career prospects (work amenity), a convenient and stable daily life (life amenity), and opportunities for personal enrichment and social belonging (cultural and study amenity). Western studies often highlight a tension between economic and cultural drivers, our results from China suggest a more integrative model.

## 1 Introduction

At present, China is undergoing a transformation from a traditional economy to a knowledge-based and innovative economy. Talent is the key to the success of the transformation. In the era of a knowledge-based economy, human resources are gradually replacing capital, land, and other production factors, which becomes the core force for promoting economic growth and enhancing urban competitiveness (1). To attract and gather high-quality talent resources, large or medium-sized cities in China have formulated and introduced a series of talent policies, including granting housing subsidies and giving municipal citizenship (2). However, these talent policies can only attract talent in the short term and have little impact on their long-term residence. Whether talents stay in the city or not is a comprehensive evaluation of the city based on their own needs, which is a complex decision-making process and is not solely influenced by talent policies. College youth are an important talent resource, and the factors influencing their willingness to stay in the city are worth exploring.

Outline of the 14th Five-Year Plan for National Economic and Social Development of the People’s Republic of China and the Long-range Goals to 2035 (an important document issued by the Chinese government to plan economic and social development) clearly puts forward concepts and goals such as “enhancing convenience and improving service experience,” “comprehensively improving urban quality,” and “improving social civilization.” These reflect that the factors of living quality, such as urban public services and social environment, have become increasingly important. At the same time, China is in a new stage of development, with per capita income and education levels generally rising (3). Therefore, youth have increasingly higher requirements for a qualitative urban living experience. It is not difficult to find that the more convenient and better the living experience of Chinese cities is in reality, the stronger the willingness of young talent to stay. By reviewing the relevant literature, we can see that the urban amenity that focuses on talent attraction could explain this phenomenon well. The theory of urban amenity is condensed in the discussion of the driving forces that promote urban development. According to this theory, the ability of attracting talents has replaced material and geographical advantages, which becomes the main driving force in promoting urban development, and urban amenity is the attraction of a city to talents (4–6).

In the era of a knowledge-based economy, urban amenities play an increasingly important role in attracting talent. Previous studies have verified the impact of urban amenity on the dwelling willingness of mobile talents (1), but it has not been extended to the field of youth talents in college. Cities in China, such as Beijing, Shanghai, Guangzhou, and Shenzhen, are the first-tier cities with the highest level of economic development, the maximum number of colleges and universities, and a large number of foreign talents, which play an important supporting and leading role in Chinese economic and social development. Improving the dwelling willingness of youth in colleges of these four first-tier cities is an important starting point for high-quality urban development. Therefore, based on the urban amenity theory, this study explored the impact of urban amenity on the dwelling willingness of college youth talents to stay in cities.

## 2 Development of urban amenity scale

Ullman (7), who first proposed the concept of urban amenity, defined urban amenity as pleasant living conditions. Considering the obvious regional nature of urban amenity (8), believed that amenity is a local feature that cannot be marketized, and this feature can attract people to live or work here. From the perspective of industrial economics, Gottlieb (9) noted that amenity is a locally specific product or service that cannot be exported, and it can benefit employees as residents or commuters. In summarizing relevant studies (10), regarded urban amenity as a composite “product” in which cities use public infrastructure and public sector workers as capital and labor input. Although scholars have different emphases on the definition of urban amenity, they all believe that urban amenity is related to people’s demand for pursuing a quality of life and has four main characteristics. First, urban amenity is regional and does not have a production function; second, urban amenity is not renewable, for example, once the wild environment is destroyed, it is irreversible; third, urban amenity is positively correlated with income, that is, it has higher income elasticity; fourth, urban amenity is generally irreplaceable (6).

Urban amenity is the core explanatory variable of this study. According to the previous literature, the measurement of urban amenity is mostly objective. Urban amenity measuring indicators are divided into four aspects: public consumer goods (such as the number of theaters per capita), aesthetic and physical environment (such as climate and beautiful buildings in the city), public service (such as schools, hospitals), and speed (such as travel traffic and distance from commercial service centers) (5). Chinese scholars divided the evaluation index of urban amenity into three categories: amenity of natural environment, amenity of service environment, and amenity of social culture (6). The amenity of the natural environment mainly refers to natural environmental conditions, including temperature, precipitation, light, atmospheric environment, water conditions, and green conditions. Amenity of service environment mainly refers to the life service environment, emphasizing artificially made, but different from nature (11), including the construction of infrastructure (such as transportation, electricity, and information technology), public services (such as schools, hospitals, and so on), and various entertainment facilities (for example, theaters, museums, cafes, and specialty restaurants). Social and cultural amenity mainly refers to the social environment and cultural landscape, including social inclusiveness, resident values, cultural atmosphere, and other related aspects. All these urban amenity evaluation indexes are objective, but lack a subjective evaluation of urban residents. Only when urban residents perceive urban amenities from daily life, work, study, and entertainment can they more truly reflect the city’s amenities. Therefore, the development of an urban amenity scale based on urban residents’ self-evaluation in accordance with rigorous and standardized procedures can not only enrich the existing urban amenity evaluation index system based on objective evaluation, but also provide a basis and reference for empirical research on urban amenity.

### 2.1 Generation of initial items

In view of the fact that the urban amenity scale should not only absorb existing studies, but also truly reflect the subjective perception of urban residents of urban amenity. This study mainly obtained the initial items of the scale through the following ways. First, we sorted out the urban amenity evaluation indexes used in previous studies and summarized the common dimensions of all urban amenity evaluation indexes. Then, 30 residents from different cities were interviewed. The topic of the interview is “What kind of city do you think is an amenity? What are the most important aspects of amenity in your city? In terms of your daily life, study, entertainment, work, and so on, what makes this city an amenity for you?” Third, through the collation and analysis of literature and interview data, 30 initial items were formed.

### 2.2 Extraction of the items

The extraction process of the initial items is as follows. First, five college students majoring in urban management were invited to combine or delete 30 initial items in a back-to-back manner. Items that are agreed to have repetitive semantics, contain multiple semantics, and are unrelated to the topic have been deleted. After discussion, a consensus was reached on inconsistent or uncertain items, and 23 items were finally retained. Second, the 5-point Likert scoring method was used to compile the items, and after discussion and modification, the initial scale of urban amenity was formed.

### 2.3 Exploratory factor analysis

In this study, a questionnaire survey was conducted among residents of major cities in China, including Beijing, Shanghai, Guangzhou, Shenzhen, Wuhan, and Hangzhou. We distributed 400 questionnaires through the “questionnaire star” platform (it is a professional online platform for questionnaire surveys and voting). When collecting the questionnaires, we found 32 invalid ones (such as random answers, incorrect answers, incomplete answers, etc.). We deleted them and eventually collected 368 valid questionnaires, with a valid questionnaire recovery rate of 92%. In the valid samples, 51.64% were male and 48.36% were female. In terms of age, 29.22% were between 19 and 23 years old, 45.59% were between 23 and 26 years old, 17.63% were between 26 and 30 years old, and 7.56% were over 30 years old. In terms of education, 19.4% held a bachelor’s degree or below, 59.7% held a master’s degree, and 20.9% held a doctorate degree.

First, the reliability of each item is assessed by the corrected item–total correlation (CITC) coefficient; the items with a CITC coefficient less than 0.5 are eliminated. Accordingly, the item “The cost of living in this city is within your acceptable range” is excluded. The Cronbach’s *α* value of the initial scale for the remaining 22 items is 0.953, indicating that the scale has good reliability. Second, Kaiser–Meyer–Olkin (KMO) and Bartlett’s sphericity tests were performed on 22 items, and the results showed that the KMO value was 0.949, and the Bartlett’s sphericity test reached a significance level of 0.001, indicating that the original data were suitable for factor analysis. Third, principal component analysis and the varimax rotation method are used for factor analysis. Factors are extracted according to the standard with an eigenvalue greater than 1, and items with a factor load value less than 0.5 are gradually removed from small to large, such as “The city’s compulsory education resources are good, the school enrollment of (future) children is convenient. The city’s climate, environment and other conditions are comfortable for you living here (less extreme weather, good air quality, and high urban greening rate).” are deleted. There are 20 items remaining on the scale. Three common factors are extracted. Exploratory factor analysis was conducted again for the remaining items. The KMO value of the sample data was 0.947, and the Bartlett’s sphericity test results were significant (*p* < 0.001). Three common factors were still extracted; the cumulative variance contribution rate reached 70.422%, and the factor load of each item was greater than 0.5. It can be seen from Table 1 that the Cronbach’s *α* coefficient of the scale as a whole is 0.952, and the Cronbach’s *α* coefficient of each factor is greater than 0.9, indicating that the scale has good reliability.

**Table 1:** Result of exploratory factor analysis.

Items	1	2	3	Cronbach’s *α*	AVE	CR	Cronbach’s *α*
There are many public study rooms in the city to facilitate your study	0.822			0.942	0.497	0.915	0.952
The city’s libraries provide you with a good learning environment	0.815						
There is a wide variety of specialty bookstores in the city that will attract you to punch in	0.799						
Students/colleagues and friends in the city are constantly learning to “charge,” “inspiring,” your own learning motivation is more sufficient	0.694						
The city or near the city’s mountains, rivers, lakes, forest pastures, ancient towns, temples, historical sites and other tourist resources are rich, you can travel long and short vacation leisure	0.693						
There are many parks in the city, and it is convenient to picnic and camp on weekends	0.685						
There are many colleges in the city, and the learning atmosphere is strong	0.682						
There are many colleges and universities in the city, and the coverage rate of national examination rooms is high, which is convenient for you to participate in various examinations	0.678						
The city’s yoga studio, gym, swimming pool, badminton hall and other sports facilities are available, which is convenient for you to exercise and fitness	0.647						
The city’s opera, crosstalk, drama, traditional drama, concert, talk show and other activities are frequent, convenient for you to choose to watch	0.621						
The city’s bars, KTV, table games, chess and card rooms and other venues are densely distributed, with large choices	0.575						
The long distance or short distance travel out of the city is convenient, there are many modes of transportation to choose from, and it is convenient		0.855		0.903	0.580	0.870	
The city is convenient to travel within the city, subway, bus, network car coverage in the city is high		0.855					
The city’s online and offline shopping experience is good, not only convenient and fast, but also thoughtful, humanized and diversified service		0.811					
The city’s catering is rich in variety, wide in taste, and strong in choice (the city’s Internet red restaurants, creative restaurants, attract you to punch the card experience)		0.702					
The medical conditions in this city make you satisfied, and it is relatively convenient to see a doctor and buy medicine and treatment		0.537					
The related industry or occupation of your major has a good development prospect in this city			0.879	0.906	0.670	0.899	
The city offers you a wider space for career advancement			0.851				
The city offers you a wealth of job options			0.823				
The salary level is higher in this city			0.764				

### 2.4 Confirmatory factor analysis

Validity testing is mainly concerned with detecting content validity and structural validity. First, the content validity of the original scale was tested by expert judgment. This scale was compiled based on a previous study on urban amenity, combined with objective indicators and the results of in-depth interviews with some urban residents, and entrusted teachers and students majoring in urban management to revise and improve the items repeatedly. Therefore, the preparation of the scale is rigorous and standardized, and the content has certain reliability. Second, the measurement of structural validity includes both convergent validity and discriminant validity. The results show that the factor load of each item is greater than 0.5, the smallest average variance extracted (AVE) value among the three factors is close to 0.5, and the combined reliability (CR) value is greater than 0.8, indicating that the scale has good convergent validity. In addition, the arithmetic square root of the three factors’ average variance extracted (AVE) value is greater than the correlation coefficient with the other factors (see Table 2), indicating that the scale has good discriminant validity.

**Table 2:** Cronbach’s *α* value, CR value, square root of AVE and correlation coefficient of each factor.

Category	F1	F2	F3
Common factor 1	0.497		
Common factor 2	0.317***	0.58	
Common factor 3	0.344***	0.305***	0.67
Square root of AVE	0.705	0.762	0.819
Cronbach’s *α*	0.942	0.903	0.906
CR	0.915	0.87	0.899

***p < 0.001; Diagonal value is factors’ average variance extracted (AVE) value.

With three common factors as latent variables, a confirmatory factor analysis test model was constructed. AMOS 26.0 structural equation software was used to analyze the structure of the three factors. Compared with the two-factor model and the single-factor model, the three-factor model had better goodness of fit (see Table 3). The absolute fitting index of the model showed: *χ*^2^/df = 2.924 (less than 3), Root Mean Square Error of Approximation (RMSEA) = 0.07 (less than 0.08). Relative fitting index (greater than 0.9) showed Comparative Fit Index (CFI) = 0.952, Normed Fit Index (NFI) = 0.929, Relative Fit Index (RFI) = 0.916, Incremental Fit Index (IFI) = 0.952, and Tucker–Lewis index (TLI) = 0.943. A reduced fitting index (greater than 0.5) yielded Parsimonious Normed Fit Index (PNFI) = 0.787, Parsimonious Goodness-of-Fit Index (PGFI) = 0.679. This indicates that the urban amenity scale is a three-dimensional global construct, and the relationship between the three factors and 20 measurement items exists and is stable.

**Table 3:** Comparison of fitness statistics for factor models.

Detection index	Absolute fit index		Relative fit index					Reduced fit index	
	CMIN/DF	RMSEA	NFI	CFI	RFI	IFI	TLI	PNFI	PGFI
Three-factor model	2.924	0.070	0.929	0.952	0.916	0.952	0.943	0.787	0.679
Two-factor model	4.639	0.096	0.887	0.908	0.867	0.909	0.893	0.756	0.646
Single factor model	11.713	0.164	0.700	0.717	0.664	0.718	0.684	0.626	0.491

The two-factor model combines items of common factor 1 and common factor 2, while the single-factor model combines items of all common factors.

### 2.5 Factors naming

The common factor (F1) contains four items, showing the advantages of jobs and salary levels provided by the city, which accurately reflect the city’s work amenities. Therefore, this factor is referred to as an urban work amenity. The common factor (F2) contains five items, showing the ease of access to transportation, medical care, education, and other resources necessary for a basic life in the city, which reflects the amenity of the city in terms of living. Therefore, this factor is referred to as an urban life amenity. The common factor (F3) contains eleven items, showing the city’s amusement facilities, leisure venues, learning spaces, and other amenities, which reflect the city’s cultural and educational amenities. Therefore, the factor is named urban cultural and study amenity. To sum up, urban work amenity refers to the city’s ability to provide high-quality employment opportunities and a favorable economic environment. Its connotation for college youth includes promising career paths, competitive salaries, a strong industrial base, and a dynamic job market. Urban life amenity refers to the ease and quality of daily living. Its connotation encompasses the accessibility and quality of practical necessities such as public transportation, healthcare, housing affordability (as noted in our limitations), safety, and retail services. Urban cultural and study amenity (revised name) refers to the city’s provision of assets that enrich intellectual, cultural, and leisure life. Its connotation for college youth is a city that is not just a place to work and live, but also a place to learn, explore, and enjoy a stimulating lifestyle.

## 3 Research hypothesis

As a measuring tool, the urban amenity scale should possess some common characteristics similar to those of other scales; otherwise, the development of the urban amenity scale will not have good application value. Therefore, it is necessary to develop a relevant model to test the application value of the scale further. This study will explore the impact of urban amenities on the willingness of college youth in first-tier cities to stay in cities, highlighting the effect of urban amenities in attracting young talent. According to the traditional definition of urban amenity (9, 12), pointed out that urban amenity refers to a series of facilities and services provided by the city that make people feel convenient, which is an immovable “local product” and welfare, and obtaining such “local product” and welfare is one of the basic motivations of population flowing into the city. The root cause of urban development and growth is the vibrant urban life and the resulting high quality of life, which attracts a diverse range of talents, especially innovative ones. Such cities and regions create an environment for people to exchange and innovate, thereby facilitating knowledge spillover (13). In other words, urban amenity is exactly what makes cities attractive to talent, and it has replaced material and geographical advantages as the main driving force for urban development (4, 5). Schmenner also pointed out that areas that can attract and retain high-quality labor will be more successful in future development, and those areas with a high quality of life and pleasant amenities would attract high-quality labor (14).

According to previous studies, when well-educated and high-quality talents choose to live and work, cities with higher amenities tend to be their first choice, which helps enhance the agglomeration of urban innovation factors and promotes sustainable urban development (15, 16). Scholars in Western developed countries have basically formed a consensus that “urban amenity and working development opportunities are the key elements of talent selection in migrating” (17–19). In recent years, China has been swept up in the wave of rapid globalization, urbanization, and the rise of a knowledge-based economy, and the study of urban amenity has attracted the attention of Chinese scholars. Especially at present, China’s economy is in a new stage of transformation, and the role of human capital and knowledge has become increasingly significant (20). As highly educated talents, college youth are important human capital, and they are scarce resources that cities compete for. The willingness of young people to stay in the city where they are studying may be influenced by the amenities of that city. First of all, young people in colleges will consider the convenience of cities more or less when choosing jobs, because cities with high-level amenities are easier to attract enterprises to settle down, and thus retain young talent (21). Therefore, the urban work amenity of this city will affect the willingness of college youth to stay in the city. Second, with the improvement of income and education level, people’s demand for urban commodity markets, services, beautiful buildings, safe living environment, convenient transportation, and other infrastructure is increasing day by day (4). Moreover, college youth tend to concentrate in cities with high artificial convenience (22). Therefore, the life amenities of the city will also affect the willingness of college youth to stay in the city to a certain extent. Third, American scholar Florida believes that cities attract talents not only by sufficient economic opportunities, high-paying jobs, and rich material convenience facilities, but also by intangible amenities such as inclusiveness, diversity, and openness (23). The People’s Daily (Chinese official media newspaper) has also summarized the reasons why Chinese young talent “flee back to Beijing, Shanghai and Guangzhou,” including the openness of big cities, more leisure places in big cities (such as cinemas, theaters, cultural centers, gyms and so on), and the inclusiveness of big cities gathering more young people with similar values. Therefore, the amenity of culture and study in the city will also affect the willingness of college youth to stay in the city.

Based on the above analysis, the following hypotheses are proposed in this study:

*H1:* Urban amenity can significantly affect the willingness of college youth in first-tier cities to stay in cities.

*H1a:* Urban work amenity can significantly affect the willingness of college youth in first-tier cities to stay in cities.

*H1b:* Urban life amenities can significantly affect the willingness of college youth in first-tier cities to stay in cities.

*H1c:* Urban cultural and study amenities can significantly affect the willingness of college youth in first-tier cities to stay in cities.

## 4 Research design

### 4.1 Research object and data source

The data used in this study are based on a random sample survey. In March 2023, an online questionnaire survey was conducted among youth from the local colleges in the four first-tier cities of China: Beijing, Shanghai, Guangzhou, and Shenzhen. The term “first-tier cities” is a common classification in China referring to the most economically and culturally advanced metropolises, and the classification is widely used in Chinese academia, media, and official reports (the classification is based on indicators like gross domestic product (GDP), population size, and so on). In the process of collecting questionnaires, we emphasized to the interviewees that the academic research questionnaires require filling in anonymously, and the results of the questionnaire would also be kept confidential. The questionnaires can only be filled in and submitted once, and they cannot be modified after submission. The selection of cities in the survey is mainly based on the following considerations: First, the above four cities are the most typical first-tier cities in China, and their economic, social, and cultural development is relatively better than that of the other cities. Discussion on their urban convenience will be more representative. Second, the above four cities are also gathering places for Chinese universities, and more youth study in these four cities. Examining the willingness of college youth in these four cities to stay in cities is also of great significance for predicting future urban development. The survey’s contents include individual characteristics, social interactions, subjective evaluations of urban convenience, and willingness to stay in the city. After removing missing value samples and invalid questionnaires, 255 valid samples were finally collected (descriptive statistics are presented in Table 4). Among them, 194 college youth expressed their willingness to stay in the city, accounting for approximately 76% of the total sample, which indicates that the college youth group has a strong willingness to stay in first-tier cities.

**Table 4:** Descriptive statistics.

Variable names	Obs	Min	Max	Mean	SD
Gender	255	0.000	1.000	0.529	0.500
Age	255	1.000	4.000	2.051	0.847
Education	255	1.000	5.000	3.051	0.659
University category	255	1.000	4.000	2.353	1.280
Marriage	255	1.000	4.000	1.090	0.371
City	255	1.000	4.000	2.341	0.999
Social relationship	255	1.000	7.000	3.702	1.405
WA	255	1.000	5.000	4.049	0.744
LA	255	1.000	5.000	4.331	0.697
CSA	255	1.000	5.000	4.178	0.671
UA	255	1.000	5.000	4.191	0.602
Willingness to stay in cities	255	0.000	1.000	0.769	0.423

UWA, urban work amenity; ULA, urban life amenity; UCSA, urban cultural and study amenity; UA, urban amenity.

### 4.2 Variable description and research method

Dependent variable: The dependent variable of this study is the willingness of college youth in first-tier cities to stay in cities, which is measured by the question, “Are you willing to stay in this city after graduation?” in the questionnaire. The answers included “yes” and “no.”

Independent variable: The key independent variable is urban amenity, which is measured by the scale developed in this study, including urban work amenity, urban life amenity, and urban cultural and study amenity.

Control variable: In comparison to previous studies, this study selects relevant control variables from the aspect of individual characteristics and social interaction. The individual characteristics mainly include the respondents’ gender, age, education level, and marital status. Social networks and social identity will both affect the settlement intention of new immigrants (24). Similarly, in this study, variables related to social network and social identity are uniformly classified as social relations and included in the control variables.

The decision of college youth on their willingness to stay in a city is a binary problem. According to the willingness to stay in a city, there are two choices: “yes” and “no,” with values of “1” and “0” respectively. Therefore, a binary logit model was employed in this study. This model is appropriate for a dichotomous dependent variable and is widely used in similar migration intention studies. The logit model was preferred over the alternative probit model primarily because its coefficients can be interpreted intuitively as odds ratios, which simplifies the discussion of results.

## 5 Result analysis

### 5.1 Reliability and validity test

In view of the fact that urban amenity is measured on a 5-point Likert scale, it is still necessary to test the reliability and validity of the data. First, Statistical Package for Social Sciences (SPSS) version 22.0 was used to conduct an exploratory factor analysis on the urban amenity data, and the KMO value was 0.925 (higher than 0.7). Bartlett’s sphericity test found *p* < 0.001, indicating that it is suitable for factor analysis. Second, the reliability test was conducted. The results showed that the Cronbach’s *α* coefficient values for urban work amenity, urban life amenity, and urban cultural and study amenity were 0.886, 0.889, and 0.933, respectively (all higher than 0.7, see Table 5). It is shown that the scales of these three variables had passed the test of internal consistency reliability. The Cronbach’s *α* coefficient for the total scale comprising three variables was 0.946, indicating that the overall structure design of the scale used in this study was highly reliable. Then, AMOS 24.0 was used to conduct confirmatory factor analysis on the recovered data. Each variable was established from a single-factor model to a three-factor model, with fitting and comparison carried out. The results show that the values of CFI, TLI and IFI in the three-factor model are 0.918, 0.905, and 0.919 (higher than 0.9), respectively, RMSEA is 0.086 (close to 0.08), *χ*^2^/df is 2.891 (less than 3), and the fit index of the three-factor model is much better than that of other factor models, which achieved a high standard. This shows that the model fits well. Finally, the validity analysis results show that the AVE value of each variable is higher than 0.5, and the CR value is higher than 0.8, indicating that the scale exhibits good convergent validity. Moreover, the square root of the AVE value of each variable is higher than its correlation coefficient with other variables, indicating that the scale has good discriminant validity.

**Table 5:** Correlation analysis, reliability and validity test of each variable.

Variable	UWA	ULA	UCSA
Urban work amenity	0.793		
Urban life amenity	0.248***	0.807	
Urban cultural and study amenity	0.275***	0.302***	0.749
AVE	0.629	0.652	0.561
CR	0.87	0.902	0.933
Cronbach’s *α*	0.886	0.889	0.933

Diagonal value is factors’ average variance extracted (AVE) value.

*, **, and *** respectively represent *P* < 0.1, *P* < 0.05, and *P* < 0.01.

### 5.2 Common method bias test

In this study, Harman’s single-factor test was used to test the Common method bias between variables. Results showed that there were four factors with an eigenvalue of more than 1, and the total variance interpretation was 71.1%. The variance interpretation of the first principal component was 25.8%, less than half of the total variance interpretation. Thus, common method bias was not a significant issue in this study.

### 5.3 Logit regression analysis

We assessed potential multicollinearity among the independent variables by calculating the Variance Inflation Factor (VIF). All VIF values were significantly below 5 (mean VIF = 1.86), indicating that multicollinearity is not a concern for the robustness of our model estimates. Then, SPSS software was used to carry out logit regression in this study. Taking control variables as the basic model, four variables—urban work amenity, urban life amenity, urban cultural and study amenity, and urban amenity as a whole—were added to the model to obtain five models, respectively. The analysis results of the models were shown in Table 6.

**Table 6:** Regression results of urban amenity and its dimensions affected on the willingness of college youth to stay in the city.

Variables	Model 1			Model 2			Model 3			Model 4			Model 5		
	*B*	SE	Exp(*B*)	*B*	SE	Exp(*B*)	*B*	SE	Exp(*B*)	*B*	SE	Exp(*B*)	*B*	SE	Exp(*B*)
Gender	0.011	0.319	1.011	−0.001	0.317	0.999	0.116	0.321	1.123	0.182	0.322	1.199	0.113	0.319	1.119
Age	−0.299	0.266	0.742	−0.226	0.265	0.797	−0.301	0.261	0.740	−0.278	0.264	0.758	−0.249	0.263	0.779
Education	0.176	0.304	1.193	0.113	0.302	1.121	0.164	0.293	1.179	0.124	0.300	1.132	0.118	0.296	1.125
College type	−0.001	0.128	1.000	−0.050	0.127	0.995	−0.050	0.130	0.951	−0.017	0.128	0.983	−0.031	0.127	0.970
Marriage	0.054	0.392	1.056	0.139	0.387	1.149	0.166	0.369	1.181	0.257	0.384	1.294	0.225	0.374	1.253
City	0.309*	0.168	1.362	0.297*	0.167	1.346	0.346**	0.167	1.413	0.386**	0.167	1.471	0.343**	0.166	1.409
Social relationship	0.81**	0.310	2.248	0.799**	0.309	2.223	0.751**	0.311	2.119	0.764**	0.312	2.148	0.762**	0.311	2.143
UWA				0.383*	0.209	1.467									
ULA							0.417**	0.120	1.518						
UCSA										0.624**	0.221	1.867			
UA													0.627**	0.244	1.871
Constant	0.143	0.916		−1.387	1.207	0.250	−1.722	1.260		−2.743**	1.334	0.064	−2.599*	1.376	0.074
Pseudo *R*^2^	0.043			0.055			0.056			0.069			0.065		
Wald-chi2	13.35*			14.55*			16.5**			20.33**			17.83**		
Observe	255														

UWA, urban work amenity; ULA, urban life amenity; UCSA, urban cultural and study amenity; UA, urban amenity.

*, **, and *** respectively represent *P* < 0.1, *P* < 0.05, and *P* < 0.01.

According to model 1, the control variables of gender, age, education level, university type, and marital status have no significant impact on the participants’ willingness to stay in the city. The variable of the city where the college youth live has passed the significance test at the 10% level in the model, indicating that compared with college youth in Beijing, those in other first-tier cities have a stronger intention to stay in the city. That is, compared with the college youth in Beijing, the willingness of college youth in other first-tier cities to stay in the city has increased by 1.362 times. The social relation variable passed the significance test at the 5% level in the model, indicating that when college youth’s friends are local residents, their willingness to stay in the city is stronger. That is, compared with the college youth whose friends are not local residents, the willingness of college youth whose friends are local residents to stay in the city is increased by 2.248 times.

Model 2 shows that urban work amenity has passed the significance test at the 10% level (*B* = 0.383; *p* < 0.1), indicating that the higher the urban work amenity, the stronger the willingness of participants to stay in the city. Compared to the urban work amenity at a low level, the higher urban work amenity increases the willingness of college youth to stay in a city by 1.467 times. Therefore, hypothesis H1a is verified. Model 3 shows that urban life amenity has passed the significance test at the 5% level (*B* = 0.417; *p* < 0.05), indicating that the higher the urban life amenity, the stronger the willingness of participants to stay in the city. Compared to the urban life amenity at a low level, a higher urban life amenity increases the willingness of college youth to stay in a city by 1.518 times. Therefore, hypothesis H1b is verified. Model 4 shows that the urban cultural and study amenities have passed the significance test at the 5% level (*B* = 0.624; *p* < 0.05), indicating that the higher the urban cultural and study amenities, the stronger the willingness of participants to stay in the city. Compared to the urban cultural and study amenities at a low level, the higher urban cultural and study amenities increase the willingness of college youth to stay in a city by 1.867 times. Therefore, hypothesis H1c is verified. Model 5 shows that the overall urban amenity has passed the significance test at the 5% level (*B* = 0.627; *p* < 0.05), indicating that the higher the urban amenity, the stronger the willingness of participants to stay in the city. Compared with urban amenity at a low level, the higher urban amenity increases the willingness of college youth to stay in a city by 1.871 times. Therefore, hypothesis H1 is verified.

## 6 Conclusion and discussion

Based on the urban amenity theory, this study discusses the impact of urban amenities in China’s first-tier cities on the willingness of college youth to stay in the city. This study first develops an urban amenity scale based on subjective cognition and evaluation of urban residents, and then establishes a binary logit regression model. Through an online questionnaire survey, first-hand data were collected to verify the model, and the main conclusions are as follows. The measurement of urban amenity can be divided into three dimensions, namely, urban work amenity, urban life amenity, and urban cultural and study amenity. Urban amenity positively affects the willingness of university students to stay in the city. Specifically, urban work amenities, urban life amenities, and urban cultural and study amenities all have a positive impact on the willingness of university students to stay in the city. In terms of control variables, the cities of college youth and the places of origin of their friends have a significant impact on their willingness to stay in the city.

Summarizing previous studies, it is found that the measurement of urban amenity is an objective indicator, which is supported by the literature of many studies. At the same time, considering the availability of objective data, the measurement of urban amenity by objective indicators can indeed gain the favor of most scholars. However, urban amenity largely depends on people’s subjective cognition and judgment (1). The urban amenity, as measured by objective indicators and data, is insufficient in terms of subjectivity. The urban amenity scale developed in this study, which mainly focuses on residents’ subjective judgment and evaluation, makes up for the lack of subjectivity of the existing measurement index system to a large extent, and introduces a new dimension of urban amenity, which is a breakthrough and improvement of the measurement index of urban amenity. The conclusion of this study aligns with the findings of Florida et al. (15). In the context of a knowledge-based economy, innovative talents, also known as the “creative class,” have a strong demand for urban amenities. Take China as an example; the main source of the so-called “creative class” is university students in first-tier cities, and their cognition and judgment of the urban amenities have actually affected their willingness to stay in the city (16).

This study finds that urban amenity positively affects the willingness of university students in first-tier cities to stay in the city. That is, the higher the level of urban amenity, the stronger the willingness of young college students to stay in the city. After graduation, college youth become a highly educated labor force, which is the core carrier of human capital (2). They are fought over by major cities, which can be seen from the “war for talent” in major cities in China. As the driving force of urban growth, urban amenity does not directly affect urban development, but makes cities more attractive to talent (25). Over the past decade, several studies on urban amenity in the Western context have shown that it has become an essential factor in attracting talent to cities (15, 16). For example, Dalmazzo and de Blasio conducted an empirical study using the household income and wealth survey data of the Bank of Italy and found that urban amenity has a strong attraction on the labor force with higher education (26). An empirical study on urban amenity in the Chinese context also shows that cities with higher amenity meet people’s needs better; thus, these cities tend to become talent attraction centers. Moreover, the correlation between urban amenity and talent is more significant, which further indicates that urban amenity is more attractive to talent (27). Therefore, the findings of this study show a pronounced effect of cultural and study amenities, challenging any assumption that non-economic factors are secondary in developing countries. This suggests that the preferences identified by Florida may be increasingly universal among the globalized, highly educated youth. Moreover, the findings of this study add nuance to the global literature by demonstrating that for Chinese college youth, amenities are not a replacement for economic concerns but a complement. The significance of all three dimensions of amenity shows that Chinese talents seek a “complete package.” It means a city that offers strong career prospects (work amenity), a convenient and stable daily life (life amenity), and opportunities for personal enrichment and social belonging (cultural and study amenity). Western studies often highlight a tension between economic and cultural drivers; our results from China suggest a more integrative model.

The study further found that the three dimensions of urban amenity—urban work amenity, urban life amenity, and urban cultural and study amenity—all positively affect the willingness of college youth in first-tier cities to stay in the city; that is, the higher the amenity of these three aspects, the stronger the willingness of youth to stay in the city. Moreover, by comparing the odds ratio of college youth’s willingness to stay in the city in first-tier cities, it is found that urban cultural and study amenity has the greatest impact on the willingness of college youth to stay in the city, second is urban life amenity, and the last is urban work amenity. Previous studies have emphasized that good educational resources, medical resources, recreational resources, and cultural and artistic services provide an amenity living environment for people, which improves the attraction of cities to the labor force, especially the highly educated labor force (2). Relevant studies by Chinese scholars emphasize the impact of basic public services and find that cities with higher quality of basic public services are more likely to attract population inflows (2, 28). For enterprises, to attract and retain talent, they need local governments to provide urban amenities such as complete infrastructural facilities and public services (2). This practice was early promoted by the early 20th-century Mayor of Birmingham, Chamberlain (who later became Prime Minister of the United Kingdom), who actively promoted municipal reform, advocated government intervention in the livelihood of the people, and provided public welfare in the city (29). After the Industrial Revolution, an urban renovation movement was initiated in Western countries, and it improved the work and living environment of the lower class, represented by the working class, which was largely driven by humanitarian reasons and a desire to guard against socialist shocks. The supply of urban public welfare has facilitated policy diffusion worldwide because of its rationality (30). The concept of “collective consumption” proposed and demonstrated by Manuel Castells, a Spanish Marxist urban theorist, is a theoretical explanation for this phenomenon (31). Since the 1960s, after the great transformation from materialism to post-materialism, some measures of urban amenity, which are based on the urban management strategy to attract the “creative class,” have gradually become universal. What’s more, this study ranks the attractiveness of urban work amenities, urban life amenities, and urban cultural and study amenities to young talent in college, which can be interpreted as that more choices and convenient access to urban entertainment places can help alleviate the academic pressure and future work pressure of college youth.

## 7 Policy implications

On the one hand, it is necessary to focus on the study of the micro-urban environment. Because urban amenity depends on people’s feelings and cognition to a certain extent, and people’s activities are more closely related to the micro-space of daily life. The study on the amenity of urban micro-environments also helps to better design urban communities and create a more effective urban space that meets people’s needs. On the other hand, China’s economy is in the transition stage from manufacturing to knowledge-intensive industries, and the demand for highly educated labor will continue to increase in the future. Additionally, with the change in consumption concept and the improvement of income level, people’s requirements for quality of life will continue to increase. Therefore, in terms of “attracting talents,” in addition to the traditional favorable policy like economic subsidies and lowering the threshold for giving municipal citizenship, in the long run, the key to attracting and retaining talents is to establish a stable labor market. At the same time, it is necessary to pay attention to improving urban quality by enhancing the configuration of public service facilities related to culture and education. Specifically, the government could provide subsidized access to museums, art galleries, theaters, and public sports facilities for youth. Funding and supporting independent bookstores, live music venues, art incubators, and innovation workshops that create a vibrant and authentic urban culture, which is particularly attractive to young people. These are the near-term, high-feasibility measures (highest priority), which face lower barriers to entry and can yield relatively quick wins. Second, the government could develop more affordable housing options for young talents or provide housing vouchers for recent graduates to alleviate their housing pressure. These are the medium-term, foundation measures, and we regarded them as the critical but more complex challenge. Third, the government could attempt to cultivate a dynamic and innovative industrial ecosystem and further improve the management system for responding to demands. On the one hand, it can provide more public services in the field of culture and art for youth, promoting urban amenity and the development of a pioneering cultural and artistic city. On the other hand, it also ensures that young people can have a better experience when accessing public services in the field of culture and art. These are all long-term and systematic measures, focusing on optimizing the overall urban construction.

## 8 Research limitations

The influence of urban amenity on the willingness of college youth talents to stay in the city is a complex issue involving multiple factors, which deserves in-depth research and exploration. Although this study attempts to be rigorous and standardized in its theoretical derivation and empirical test, it still has the following deficiencies due to limited research experience and resources. First, in terms of data, this study is based solely on the sample data of a random sampling survey, and the sample size is not large enough, which may lead to bias in the research results. The data have passed the reliability and validity tests, as well as the common method bias test. However, future studies can further expand the sample size to improve the reliability and validity of the study. Second, in terms of control variables, the omission of a socioeconomic variable (such as parental income or family wealth) is a substantial limitation. Given that housing affordability is arguably the single greatest barrier to long-term settlement in first-tier cities, this omission means our model cannot account for a major source of variation in retention willingness. This likely leads to an overestimation of the net effect of amenities for a significant portion of the population. We strongly recommend that future studies prioritize the inclusion and analysis of such covariates. Third, in terms of the generalizability of our findings, they may be limited by the unique context of Chinese first-tier cities. These cities represent an extreme case of economic development, high living costs, and intense competition. The relative importance of work, life, and cultural amenities likely varies across different city tiers and cultural contexts. Generalizing the findings requires caution, and future comparative research across different city tiers and national contexts is needed to build a universal theory. Furthermore, our findings must be interpreted within the unique institutional context of China’s Household Registration (Hukou) system. While our “urban life amenity” dimension captures perceptions of healthcare and other public services, access to these services, especially in first-tier cities, is often contingent upon obtaining a local Hukou. The high cost of living and the difficulty of acquiring a Hukou in cities like Beijing and Shanghai present a formidable structural barrier that may attenuate the positive relationship between amenity perceptions and retention willingness (32). Future research should explicitly integrate Hukou-related variables to disentangle the complex interplay between soft amenities and hard institutional constraints in shaping the futures of China’s educated youth.

## Data Availability

The raw data supporting the conclusions of this article will be made available by the authors, without undue reservation.
